# Establishment of a Prediction Model to Diagnose the End-stage Knee Osteoarthritis Based on a Significant Difference in Ferroptosis-Related Genes in Chondrocytes

**DOI:** 10.1055/a-2762-1558

**Published:** 2026-01-20

**Authors:** Lingtian Min, Cheng Chen, Weijun Wang

**Affiliations:** 1 Department of OrthopedicsNantong Hospital to Nanjing University of Chinese MedicineNantongChina; 2 Department of OrthopedicsThe Suqian Clinical College of Xuzhou Medical UniversitySuqianChina; 3 Department of Orthopedics 66506 Nanjing University Medical School, Affiliated Nanjing Drum Tower HospitalNanjingChina

**Keywords:** ferroptosis, knee osteoarthritis, diagnostic model, random forest, supervised machine learning, Ferroptose, Knieosteoarthrose, diagnostisches Modell, Random Forest, überwachtes maschinelles Lernen

## Abstract

**Background:**

Knee osteoarthritis (OA) is a widespread joint disease with no disease-modifying treatments. Chondrocyte damage is a key process in knee OA and ferroptosis is lipid peroxidation-induced iron-dependent cell death that exacerbates the process of knee OA and aggravates an imbalance in the synthesis as well as degradation of matrix metallopeptidase 13 (MMP13) and type II collagen. The clinical diagnosis of knee OA mainly depends on imaging. Whether ferroptosis-related genes could be used as new biomarkers for the diagnosis of OA remains to be explored.

**Methods:**

A dataset was used to build a diagnostic model used to diagnose and differentiate patients with end-stage knee OA. Normalization and quality control of the three profiles was carried out using R 4.1.0.

**Results:**

Analysis of a dataset (GSE114007) of differentially expressed genes (DEGs) found that the expression of 15 ferroptosis-related genes, including activating transcription factor 3 (ATF3), cyclin-dependent kinase inhibitor 1A (CDKN1A), and cytochrome b-245 beta chain (CYBB), showed significant changes in osteoarthritic chondrocytes relative to normal subjects. Based on 15 ferroptosis-related genes, we developed and compared diagnostic models using different supervised learning algorithms.

**Conclusions:**

The diagnostic model based on the support vector machine gave a convincing diagnostic performance for both verifications (Area Under Curve [AUC] = 0.9601) and testing (AUC = 0.8725). The results collectively indicate that ferroptosis-related genes may play an indispensable role in knee OA and could be specific diagnostic biomarkers for knee OA.

## Zusammenfassung

**Hintergrund:**

Die Knieosteoarthrose (OA) ist eine weitverbreitete Gelenkerkrankung ohne krankheitsmodifizierende Therapien. Chondrozytenschädigung ist ein zentraler pathologischer Prozess bei Knie-OA. Ferroptose – eine neuartige, eisenabhängige Zelltodform, die durch Lipidperoxidation induziert wird – verschärft das Fortschreiten der Knie-OA und stört das Gleichgewicht zwischen Synthese und Abbau von Matrix-Metallopeptidase 13 (MMP13) sowie Kollagen Typ II. Die klinische Diagnose von Knie-OA hängt hauptsächlich von bildgebenden Methoden ab. Ob ferroptoseassoziierte Gene als neuartige diagnostische Biomarker für Knie-OA dienen können, bleibt noch zu untersuchen.

**Methoden:**

Der Datensatz wurde verwendet, um ein Modell zur Diagnose und Differenzierung von Patienten mit terminaler Knie-OA zu erstellen. Die Normalisierung und Qualitätskontrolle der 3 Profile wurden mit der Software R 4.1.0 durchgeführt.

**Ergebnisse:**

Die Analyse eines Datensatzes mit differenziell exprimierten Genen (DEGs, GSE114007) ergab, dass 15 ferroptoseassoziierte Gene – darunter Activating Transcription Factor 3 (ATF3), Cyclin Dependent Kinase Inhibitor 1A (CDKN1A) und Cytochrom B-245 Beta Chain (CYBB) – im Vergleich zu Gesunden bei osteoarthritischen Chondrozyten signifikante Expressionsveränderungen aufwiesen. Auf Basis der 15 ferroptoseassoziierten Gene wurden diagnostische Modelle mit verschiedenen überwachten Lernalgorithmen erstellt und verglichen.

**Schlussfolgerungen:**

Das diagnostische Modell auf Basis der Support-Vektor-Maschine zeigte eine überzeugende diagnostische Leistung sowohl in der Validierung (Area Under Curve [AUC] = 0,9601) als auch im Test (AUC = 0,8725). Zusammengefasst weisen diese Ergebnisse darauf hin, dass ferroptoseassoziierte Gene eine unverzichtbare Rolle bei Knie-OA spielen könnten und als potenzielle spezifische diagnostische Biomarker für Knie-OA dienen könnten.

## Introduction

Osteoarthritis (OA) is a common chronic degenerative joint disease that affects people worldwide 1 . As the aging process accelerates worldwide and the number of obese individuals increases, the prevalence of OA is projected to rise 2 . From a clinical perspective, the knee joint is the most common site affected by OA 3 . Knee OA is responsible for around 85% of the global burden of OA 4 . Accumulating evidence indicates that age, gender, trauma, and obesity are significant risk factors for knee OA 5 . Patients with knee OA suffer from pain and disability, for which there are neither cures nor disease-modifying treatments 6 . OA places a heavy burden on the economy and health of both older adults and societies. Knee OA is a complex disease involving the entire joint structure.

The loss of articular cartilage, subchondral bone thickening, osteophyte formation, and synovial inflammation are the core pathological features of knee OA 7 . But first and foremost, knee OA is characterized by the degradation of articular cartilage 8 . During the pathogenesis of knee OA, the composition of articular cartilage undergoes alterations and loses its structural integrity 9 . Damage to cartilage integrity results in the joint’s resistance to external forces being weakened, which makes it more vulnerable to injury. Chondrocytes are the sole cell component in articular cartilage, and they maintain the integrity of the extracellular matrix (ECM) by balancing the synthesis and breakdown of ECM components 10 . In the context of knee OA, the dysregulation of matrix metallopeptidase 13 (MMP13) and matrix metallopeptidase 3 (MMP3) plays a crucial role in disease progression. Chondrocyte homeostasis is indispensable for preserving normal joint function and preventing knee OA. The clinical manifestations of knee OA include pain, stiffness, decreased joint movement, and muscle weakness 2 11 .

To date, the clinical diagnosis of knee OA is mainly based on imaging 12 . Plain radiography helps confirm the diagnosis of knee OA by detecting the characteristics of knee OA, including joint space narrowing, osteophyte formation, subchondral sclerosis, and cysts 13 14 . The biochemical indicators used for the assessment of knee OA patients are mainly C-reactive protein (CRP) levels and the erythrocyte sedimentation rate (ESR), which can reflect the disease severity of knee OA. The pathological examination of articular cartilage plays an essential role in determining the disease classification. However, the development of knee OA occurs gradually. Personal health management should be initiated at an early stage, with conservative treatment as the primary intervention. In the middle stage, natural joint reconstruction should be the primary goal, including high tibial osteotomy (HTO), fibula osteotomy (FO), etc.; these operations preserve the patient’s cartilage as far as possible and provide the basis for a natural reconstruction of the joint. End-stage knee OA falls in the category of total knee arthroplasty (TKA). It is therefore inappropriate to simply expand the indications for surgery. Instead, treatment should follow the stepwise progression of knee OA, and a staged therapeutic approach should be adopted to avoid premature joint reconstruction in cases that could have achieved natural recovery. The aim is to prevent unnecessary prosthetic replacement. The key to staged treatment lies in an accurate evaluation of joint status. This means that a more precise detection strategy to identify whether knee OA patients have progressed to end-stage OA is urgently needed.

Chondrocytes decrease with the progression of knee OA. Chondrocyte status thus determines whether articular cartilage tissue can be rebuilt naturally or needs to be replaced entirely. Recently, studies have shown that ferroptosis is related to many age-related diseases 15 . Previous studies have demonstrated that knee OA is associated with certain aspects of iron deposition, such as abnormal iron metabolism 16 , lipid peroxidation 17 , and mitochondrial dysfunction 18 . Intra-articular injection of ferrostatin-1, a specific inhibitor of ferroptosis, can rescue the protein expression of GPX4 and type II collagen and alleviate cartilage degeneration in patients with temporomandibular joint osteoarthritis 19 . Interleukin (IL)-1β and ferric ammonium citrate (FAC) can induce the accumulation of lipid reactive oxygen species (ROS) and alterations in the expression of ferritin-related proteins, leading to the death of chondrocytes. Chondrocyte ferroptosis exacerbates the process of knee OA and aggravates the imbalance in the synthesis and degradation of MMP13 and type II collagen 20 . A feasible strategy to evaluate osteoarthritis could be the detection of ferroptosis-related genes. As pathological examinations can be used to detect chondrocytes, they should assist in the assessment of knee OA. However, whether ferroptosis-related genes can be used as new biomarkers for the diagnosis of end-stage knee OA remains to be explored.

## Materials and Methods

### Knee OA gene expression profiles procurement in cartilage tissue of the knee joint

As is the case with deoxyribonucleic acid (DNA), the genetic information in messenger ribonucleic acid (mRNA) is encoded in nucleotide sequences, and mRNA can serve as a template for protein synthesis. While mRNAs act as transient intermediate molecules in information transmission networks, noncoding RNAs have distinct, alternative functions. The transcriptome captures a temporal snapshot of the total transcripts present in a cell. Transcriptomic techniques provide a comprehensive overview of cellular processes. Based on existing transcriptome data of knee joints from clinical patients with knee OA and healthy individuals, we can further mine the transcriptome data and obtain novel findings with bioinformatics. Two datasets were retrieved from the Gene Expression Omnibus (GEO) database. All datasets were tested on the GPL960 microarray probe platform (GSE114007 and GSE117999). GSE114007 was used for machine learning as a training and validation set, while GSE117999 served as a test set to eliminate possible interferences between gene expression profiles. Patients in both datasets were end-stage knee OA patients undergoing knee replacement. The dataset was used to construct a diagnostic model for diagnosing and differentiating end-stage knee OA patients. Normalization and quality control of the two profiles were carried out with R 4.1.0.

### Differentially expressed genes and functional enrichment analysis of cartilage tissue of the knee joint

The data from GSE114007 and GSE117999 were normalized using the quantile method of the limma R package, and DEGs were screened using threshold values of FC > 1.5 and p < 0.05. Differentially expressed genes (DEGs) were filtered with the stringr and limma packages for R 4.1.0. Gene expression in the knee OA and control groups was assessed based on the fold change (FC) in the cartilage tissue of the knee joint. The DEGs were identified with the cutoff values (FC > 0.512 and p < 0.05). DNA probe IDs corresponding to these genes were matched with their gene symbol. Hierarchical clustering and expression difference of DEGs were visualized and analyzed using the pheatmap package of R.

DEGs were subjected to gene ontology (GO) enrichment analysis for functional annotation, encompassing biological process (BP), cell composition (CC), and molecular function (MF). Kyoto Encyclopedia of Genes and Genomes (KEGG) analysis was performed to explore the biological signaling pathways associated with DEGs.

### Differential ferroptosis-related genes for knee OA

Ferroptosis-related genes were retrieved from the FerrDb database ( http://zhounan.org/ferrdb ). The Venn diagram package was used to compare DEGs and ferroptosis-related genes. The String database was used to construct a protein-protein interaction (PPI) network of ferroptosis-related genes in knee OA. Principal component analysis (PCA) with the ggfortify package was additionally used to distinguish knee OA samples from control samples based on ferroptosis genes related to knee OA. A box plot of knee OA genes related to ferroptosis was depicted with the complot and tidyverse packages.

Fourteen key genes related to knee OA, validated by previous studies, were obtained by consulting the literature 21 . The Venn diagram package was used to visualize the intersections between DEGs and these 14 key genes, which included MMP13, collagen type I alpha 1 chain (COL1A1), collagen type II alpha 1 chain (COL2A1), and collagen type III alpha 1 chain (COL3A1). PCA was performed to distinguish knee OA samples from control samples based on key genes related to knee OA.

### Machine learning analysis in a diagnostic prediction model

As part of the development of a diagnostic model for detect end-stage knee OA, ferroptosis-related DEGs were identified as independent variables. The GSE114007 dataset was used as both the validation and training set, while the GSE117999 dataset served as the test set. Feature collection was performed using the sklearn.model selection in Python. Disparate algorithms were implemented to investigate a potential diagnostic model for knee OA based on ferroptosis-related genes. The support vector classification (SVC) and random forest (RF) models were constructed with the sklearn.svm and sklearn.ensemble libraries. The SVC model exhibited superior performance compared to the RF model. Receiver Operating Characteristic (ROC) curves were plotted using the matplotlib library to assess the diagnostic performance of the models. The diagnostic model was first validated using the validation set, and subsequently evaluated again with the test set to verify its applicability to external datasets.

At the same time, we established an additional diagnostic prediction model for knee OA based on the gene expression levels of MMP13, COL1A1, COL2A1, and COL3A1 in the cartilage tissue of the knee joint. By comparing its predictive accuracy with that of the ferroptosis-related diagnostic prediction model, we further validated the reliability and accuracy of the latter.

## Results

### Validation and functional analysis of knee OA DEGs in cartilage tissue of the knee joint

**Fig. 1:**
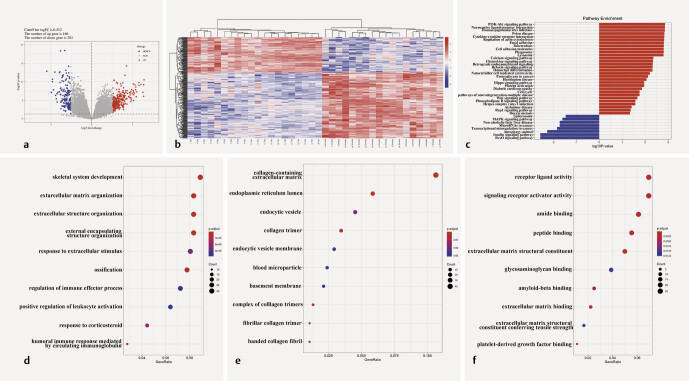
Functional and validation analysis of DEGs in knee OA. **a** DEGs’ (FC > 1.5 and p < 0.05) cutoff values for acute myocardial infarction knee OA and the control group (red points are upregulated genes; blue points are downregulated genes). **b** All of the DEGs and clinical status hierarchical clustering. **c** KEGG pathway analysis for the DEGs. **d** – **f** GO enrichment analysis of DEGs, including BP, CC, and MF, as well as the KEGG pathway of the DEGs.

**Fig. 2:**
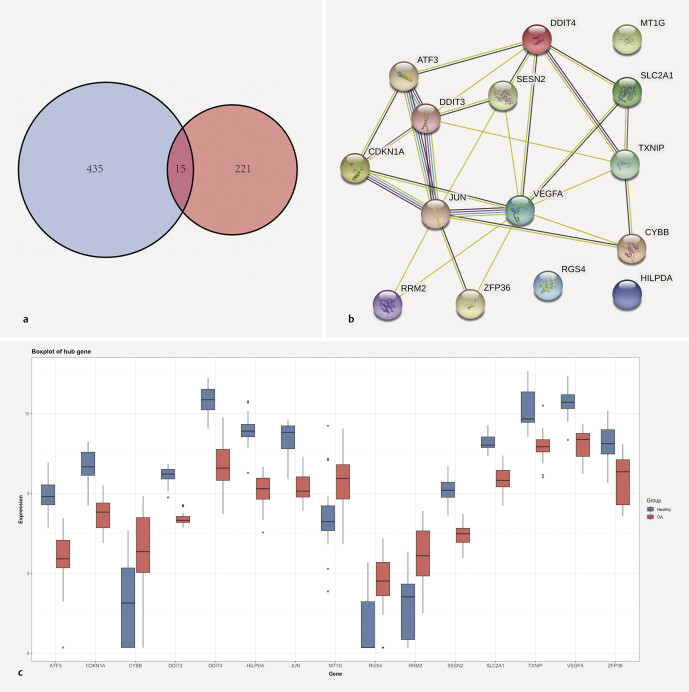
Expression and functional analysis of genes related to differential iron death in knee OA. **a** The intersection between the collected ferroptosis-related genes and the differentially expressed genes (DEGs) in knee OA. **b** The protein-protein interaction (PPI) network of these iron-death-related genes; and ( **c** ) the expression of different ferroptosis-related genes between the knee OA group and the control group, using two-tailed Student’s t-test (p < 0.05) is significant).

The baseline characteristics of all participants are presented in Table 1 . The 38 samples in the GSE114007 (knee OA and controls) dataset were normalized to adjust the gene expression values measured with different conditions to a notionally common scale. Fold change (FC) values were calculated using the limma package. Based on the cutoff values (|FC| > 1.5 and p < 0.05), 450 differentially expressed genes (DEGs) were identified in knee OA and control groups, including 166 upregulated genes and 284 downregulated genes ( Fig. 1 **a** ). To further explore the pathophysiological roles of these DEGs in knee OA relative to controls, GESA pathway enrichment analysis showed that the top two significantly enriched signaling pathways (with the smallest p-values) for upregulated and downregulated DEGs were the PI3K-Akt signal pathway, neuroactive ligand-receptor interaction, the FoxO signaling pathway, and the insulin signaling pathway ( Fig. 1 **c** ). In addition, BP ( Fig. 1 **d** ), CC ( Fig. 1 **e** ), and MF ( Fig. 1 **f** ) annotations were clustered based on GO functional enrichment analysis. As a result, most genes were involved in bone growth as well as the extracellular matrix and structure in BP, and CC was enriched in collagen-related biological functions, while MF was enriched in receptor activity-related functions. More importantly, hierarchical clustering was performed to verify the reliability of the DEGs. and the heatmap clearly distinguished the control and knee OA groups ( Fig. 2 **b** ).

**Table:** **Table 1** Baseline characteristics of patients.

Characteristics	GSE114007			GSE117999		
	Normal	OA	p value	Normal	OA	p value
N	18	20	–	12	12	–
Age, mean ± SD	36.611 ± 13.461	66.2 ± 7.3456	< 0.001	49.167 ± 10.25	65.25 ± 7.9444	0.0003
Gender, n (%)	–	–	0.058	–	–	0.214
Female	5 (13.2%)	12 (31.6%)	–	5 (20.8%)	9 (37.5%)	–
Male	13 (34.2%)	8 (21.1%)	–	7 (29.2%)	3 (12.5%)	–
Grade, n (%)	–	–	< 0.001	–	–	–
1	18 (47.4%)	0 (0%)	–	–	–	–
4	0 (0%)	20 (52.6%)	–	–	–	–
BMI, mean ± SD	–	–	–	26.897 ± 3.9085	36.273 ± 6.6036	0.0005

### Differential ferroptosis-related genes of knee OA in cartilage tissue of the knee joint

By comparing the DEGs with ferroptosis-related genes, 15 differentially expressed ferroptosis-related genes were obtained. These included activating transcription factor 3 (ATF3), cyclin-dependent kinase inhibitor 1A (CDKN1A), cytochrome B -245 beta chain (CYBB), DNA damage-inducible transcript 3 (DDIT3), DNA damage-inducible transcript 4 (DDIT4), hypoxia-inducible lipid droplet associated (HILPDA), JUN proto-oncogene, AP-1 transcription factor subunit (JUN), metallothionein 1 G (MT1G), regulator of G protein signaling 4 (RGS4), ribonucleotide reductase regulatory subunit M2 (RRM2), sestrin 2 (SESN2), solute carrier family 2 member 1 (SLC2A1), thioredoxin interacting protein (TXNIP), vascular endothelial growth factor A (VEGFA), and ZFP36 ring finger protein (ZFP36; Fig. 2 **a** ). Specifically, these 15 ferroptosis-related genes were significantly differentially expressed in the cartilage tissues of patients with knee OA, with a p-value < 0.05 ( Fig. 2 **c** ).

The results indicated that these ferroptosis-related genes may play a potential role in the pathogenesis of knee OA and chondrocyte degeneration.

Meanwhile, a PPI interaction network of the DEGs of ferroptosis-related proteins was constructed using the STRING database. ( Fig. 2 **b** ). As reported previously, JUN and VEGFA could be the hub genes among these 15 ferroptosis-related genes, given their interactions with the other 13 genes in the network.

### Construction of a diagnostic model for knee OA using differential ferroptosis-related genes

**Fig. 3:**
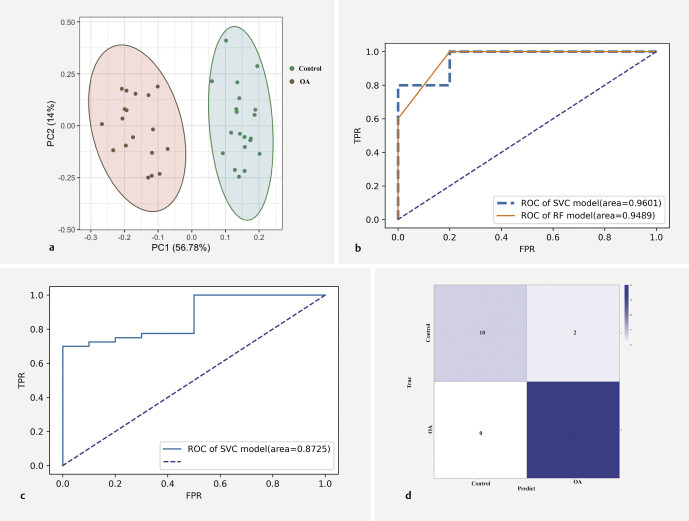
Construction of an RF diagnostic model for knee OA through differential ferroptosis-related genes. **a** PCA of the differentially expressed genes related to ferroptosis for dimensionality reduction. **b** Two different supervised learning model comparisons; and ( **c** – **d** ) the predictive model was tested to evaluated the diagnostic performance (ROC and confusion matrix).

PCA was employed as a dimensionality reduction strategy based on the differentially expressed genes related to ferroptosis. The PCA results demonstrated that the two groups could be accurately distinguished ( Fig. 3 **a** ), indicating that these genes can serve as independent characteristic parameters for the diagnosis of knee OA. Furthermore, the GSE114007 dataset, comprising 20 knee OA patients and 18 healthy individuals, was divided into a training set and a validation set. The GSE117999 dataset, which included 12 knee OA patients and 12 healthy individuals, was used as an independent test set. Two supervised machine learning algorithms (SVC and RF) were utilized to construct a diagnostic prediction model for knee OA. In this study, the diagnostic accuracy of the SVC model (accuracy = 0.95) was higher than that of the RF model (accuracy = 0.90). Moreover, the ROC curve of the GSE114007 dataset (validation set) also showed that the area under the curve (AUC) of the SVC model was 0.9601, which was better than the RF model’s AUC of 0.9489 ( Fig. 3 **b** ). Collectively, a comprehensive analysis of these metrics indicated that the SVC model enabled the most accurate diagnosis of knee OA patients, thereby providing a valuable opportunity for early intervention. Consequently, the SVC algorithm was selected for the further construction of a knee OA diagnostic model in this study.

Meanwhile, the GSE117999 dataset was designated an independent test set to externally validate a diagnostic model based on the SVC. As shown in Fig. 3 **c** , the ROC curve was validated with external data (AUC = 0.8725), demonstrating that the knee OA diagnostic model established by the SVC exhibits excellent diagnostic performance. More importantly, the classification model was visually evaluated with the confusion matrix ( Fig. 3 **d** ). All twelve knee OA patients were correctly diagnosed, while two healthy individuals were falsely classified as having knee OA. Notably, no knee OA patients were misdiagnosed as healthy volunteers, indicating that the diagnostic model could effectively reduce the false negative rate.

### Construction of a diagnostic model based on the differences in MMP13, COL1A1, COL2A1, and COL3A1

**Fig. 4:**
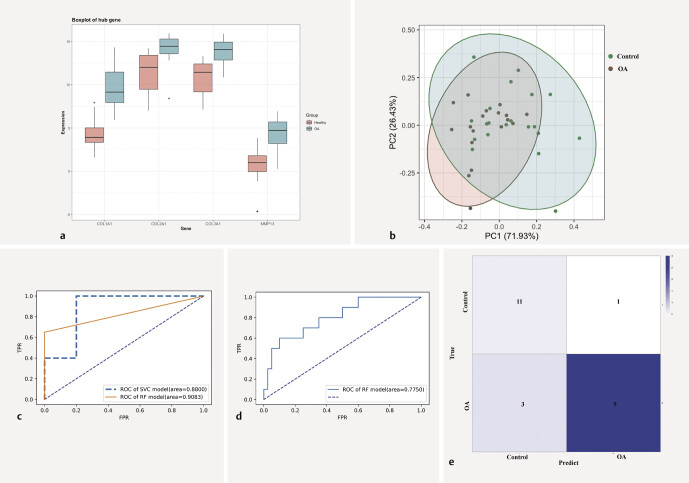
An RF diagnostic model for knee OA based on differential ferroptosis-related genes. **a** PCA of the differential expressions of MMP13, COL1A1, COL2A1, and COL3A1 for dimensionality reduction. **b** Comparison of two different types of supervised learning models (RF and SVC); and ( **c** – **d** ) the predictive model was tested to evaluated the diagnostic performance (ROC and confusion matrix).

To further investigate whether ferroptosis-related genes offer greater advantages than traditionally verified knee OA marker genes, we identified 14 established knee OA marker genes via a literature search. After a comparison of these 14 genes with the DEGs, four genes–MMP13, COL1A1, COL2A1, and COL3A1–were selected as key genes for subsequent research ( Fig. 4 **a** ). Four genes were significantly upregulated in the cartilage tissue of knee OA patients, with a p-value of < 0.05 ( Fig. 4 **b** ). However, PCA’s dimensionality reduction processing with the four genes revealed that, compared with ferroptosis-related genes, MMP13, COL1A1, COL2A1, and COL3A1 were less effective at distinguishing between OA and control groups ( Fig. 4 **c** ). Correspondingly, we established a knee OA diagnosis model using these four genes, based on the SVC and RF models. The diagnostic accuracy of the SVC model (accuracy = 0.82) was lower than that of the RF model (accuracy = 0.87). The ROC curve of the validation set of GSE117999 dataset also showed the AUC of the SVC model (0.8800) to be lower than that of the RF AUC (0.9083; Fig. 4 **d** ). Therefore, the RF model, with its superior performance, was selected for subsequent investigations.

The GSE117999 dataset was used to externally verify the diagnosis model through the RF algorithm. Fig. 4 **e** shows the ROC curve (AUC = 0.7750) verified by external data, which indicated that the knee OA diagnostic model established by the RF algorithm has a better diagnostic performance. More importantly, the classification model was visually assessed using the confusion matrix ( Fig. 4 **f** ). Eight knee OA patients, as a model group, were correctly classified, and eleven healthy volunteers were also accurately identified. However, three knee OA patients were misclassified as healthy individuals, indicating that the method had a false negative rate.

## Discussion

Knee OA is recognized as the most common global chronic joint disease. As populations are aging accompanied by a global obesity epidemic, the incidence of osteoarthritis is on the rise. Knee OA imposes a substantial burden on patients, families, and society in general, making the standardized diagnosis and treatment of this condition crucial in clinical practice. The core goals of knee OA treatment include relieving pain, slowing disease progression, improving or restoring joint function, correcting deformities, and enhancing patients’ quality of life. Therefore, a concept of a stepwise therapy has emerged, which consists of basic therapy, drug therapy, repair therapy, reconstructive therapy, and finally, TKA surgery 22 .

During restorative treatment, various types of surgery, including microfracture surgery and arthroscopic chondroplasty 23 , can be used to assess the status of diseased articular cartilage and determine the severity of knee OA in a patient. We established an OA diagnostic model based on the transcriptomics of cartilage tissue from patients with end-stage OA, which can identify whether a patient’s cartilage is capable of natural regeneration or requires complete replacement with a prosthesis. Previous studies have reported that numerous synovial fluid biomarkers in knee OA, including VEGF, Leptin, MMP-1/3, and tissue inhibitor of metal protease 1 (TIMP-1), can be realistically used in clinical practice 24 . The molecular biomarkers in serum or synovial fluid have been reported to be predictive of knee OA progression, offering potential clinical utility for early risk stratification of asymptomatic individuals and monitoring patient responses to disease-modifying interventions 25 .

In the present study, 15 ferroptosis-related genes were screened from 452 significantly differentially expressed genes. PCA results showed that these 15 genes can significantly distinguish knee OA patients from healthy individuals, indicating the presence of significant alterations in these ferroptosis-related genes in chondrocytes of end-stage OA. To establish a diagnostic model for evaluating the severity of knee OA, we constructed a model of 15 ferroptosis-related genes that were significantly changed in chondrocytes using multiple machine learning approaches. Results from the SVC and RF models demonstrated that ferroptosis-related genes can be used to develop a diagnostic model which would provide a more accurate forecast of progression to end-stage knee OA. Notably, ATF3 plays a regulatory role in the expression of inflammatory cytokines in chondrocytes which is closely implicated in the occurrence and development of OA 26 . Moreover, recent research has shown that the JNK-JUN signaling axis modulates the expression of nuclear receptor coactivator 4 (NCOA4) and has a pivotal regulatory effect on chondrocyte ferroptosis as well as on the pathogenesis of OA 27 . VEGFA is associated with superior postoperative outcomes following unicompartmental knee arthroplasty (UKA), and this association may offer a clinically applicable tool for optimizing patient selection and enhancing prognostic evaluation in UKA practice 28 . Furthermore, CDKN1A expression has previously been reported to be downregulated in chondrocytes derived from OA cartilage compared to those from normal cartilage tissue 29 . To further demonstrate that the diagnostic advantage stems from the ferroptosis-related gene set itself, rather than algorithm selection, we compared and analyzed the model constructed with established traditional marker genes (MMP13, COL1A1, COL2A1, and COL3A1). The results clearly indicate that the model based on iron death exhibits excellent accuracy and stability, highlighting the greater diagnostic value of iron death gene features.

In summary, we established a diagnostic method that can accurately identify whether patients have progressed to end-stage knee OA, based on the biological mechanism of chondrocyte ferroptosis. Specifically, this method can be used to determine whether patients are suitable for minimally invasive surgical repair combined with natural cartilage regeneration (HTO and FO, etc.) or if TKA surgery is the only viable therapeutic alternative. However, the applicability of the diagnostic model established by ferroptosis-related genes needs further clinical research.

## Conclusions

We identified 15 ferroptosis-related genes in patients with knee OA and verified their potential functions using the GSE114007 and GSE117999 datasets. After evaluating various supervised learning models on the knee OA gene dataset, an SVC model was established based on the DEGs related to ferroptosis in knee OA (AUC = 0.9601) through K-fold cross-validation, which was further validated with an external dataset (AUC = 0.8725).
