# Correction: Establishment of a Prediction Model to Diagnose the End-stage Knee Osteoarthritis Based on a Significant Difference in Ferroptosis-Related Genes in Chondrocytes

**DOI:** 10.1055/a-2798-1014

**Published:** 2026-02-02

**Authors:** Lingtian Min, Cheng Chen, Weijun Wang

**Affiliations:** 1 Department of OrthopedicsNantong Hospital to Nanjing University of Chinese MedicineNantongChina; 2 Department of OrthopedicsThe Suqian Clinical College of Xuzhou Medical UniversitySuqianChina; 3 Department of Orthopedics 66506 Nanjing University Medical School, Affiliated Nanjing Drum Tower HospitalNanjingChina



In the above-mentioned article the funding information was not correct. Correct is: 2024JZ038 | the Special Program for Clinical Medicine of Nantong University Z2023045 | the Medical Research Foundation of Jiangsu Provincial Health Commission 20210290 | the Medical Research Foundation of Jiangsu Provincial Health Commission This was corrected in the online version on February 2, 2026.

